# Risk Factors Associated with Carious Lesions in Permanent First Molars in Children: A Seven-Year Retrospective Cohort Study

**DOI:** 10.3390/ijerph17041421

**Published:** 2020-02-22

**Authors:** Carmen Llena, Elena Calabuig, José Luis Sanz, Maria Melo

**Affiliations:** Department of Stomatology, University of Valencia, Gascó Oliag 1, 46010 Valencia, Spain; elenacalabuig@hotmail.com (E.C.); jsanzalex96@gmail.com (J.L.S.); maria.melo.alminana@gmail.com (M.M.)

**Keywords:** dental caries, molar incisor hypomineralization, permanent first molar, caries risk factors

## Abstract

The aim of this study was to evaluate the risk factors associated with the occurrence of caries in permanent teeth (PT) and in the permanent first molar (PFM) seven years after their eruption. Children born in 2005 who were enrolled in a Community Dental Program were included. A total of 278 children were enrolled. Evaluated risk factors were parental caries experience, educational level of the mother, routine medications, systemic diseases, dietary habits, toothbrushing frequency, existence of molar incisor hypomineralization (MIH) in the PT, and caries in the temporary teeth (TT). Associations between independent variables and the DMF-T (decayed, missing, and filled teeth in PT) and DMF-M (DMF in PFM) indices, only considering cavitated and non-cavitated carious lesions or cavitated carious lesions as outcomes, were evaluated by Poisson regression with robust variance analysis. A cariogenic diet (sweets and soft drinks), toothbrushing frequency of <1 a day, a presence of df-t (decayed and filled temporary teeth) score of >0, low educational level of the mother, and existence of MIH were associated with high DMF-T or DMF-M values (*p* < 0.05). We can conclude that the intake of sweets and soft drinks, toothbrushing frequency, the presence of caries in TT, and MIH in PT were the best predictors of the occurrence of caries in PT and PFM.

## 1. Introduction

Dental caries are the most prevalent non-transmissible disease in humans [1]. Carious lesions appear as a result of a change in the ecology and metabolic activity of the biofilm, which results in pH fluctuations that can lead to the sub-superficial demineralization of enamel [2].

The interaction between acidogenic microorganisms, sugar attacks, and host susceptibility are responsible for the development of new carious lesions and the progression of existing ones [3]. Other factors, such as toothbrushing with a fluoridated toothpaste, or behavioral, social, and cultural factors influence the progression of caries both positively and negatively [4,5].

Epidemiological studies have facilitated the determination of the prevalence, incidence, and distribution of dental caries in different countries and regions, as well as the evaluation of the impact of preventive and therapeutic measures applied in oral public health [6,7,8]. National and regional epidemiological studies carried out in Spain have demonstrated that caries prevalence in temporary teeth (TT) at 5–6 years of age has increased in the last 5 years, while in permanent teeth (PT) a noticeable reduction at 12 and 15 years of age has been noted in the last 20 years [9,10].

In clinical practice, the use of risk criteria for dental caries helps in identifying the patients with the highest probability of developing the disease during a certain period of time, as well as the individuals in which the disease will progress or arrest [11,12]. Multivariate models display better accuracy than the use of single predictors, especially for preschool children [11,12,13,14].

The range of 5 to 13 years of age covers a long period in which both dentitions coexist and share potentially cariogenic exposures [15]. The positive relationship between dental caries in TT and PT is well documented [16]. For example, the presence of caries in the distal surface of the second temporary molar increases the risk of caries in the mesial surface of the permanent first molar (PFM) [17,18]. Occlusal surfaces of PFMs and the buccal pit in permanent mandibular first molars represent the most frequent locations for the occurrence of caries during the period of mixed dentition [19,20]. Consequently, they often require specific preventive measures or restorative treatments, or may be lost prematurely due to caries.

The preservation of PFM health is a clear determining factor for future dental health. The age at eruption, position, and difficulty for adequate plaque removal, together with the fact that parents are often unaware of the permanent nature of this tooth, make it particularly vulnerable to caries [21].

The increasing incidence of molar incisor hypomineralization (MIH) constitutes a risk factor for the vulnerability of the PFM, as it results in enamel fracture and an increase in the incidence of caries, as well as the presence of moderate or severe hypersensitivity. Therefore, affected molars require early diagnosis and adequate preventive/therapeutic measures to avoid their loss [22].

In a previous study, carried out by our research group, risk factors included in the dental clinical history (DCH) and their association with the occurrence of caries in PT—particularly in the PFMs of the same population cohort—5 years after its eruption were assessed. The conclusions were that the intake of sweets and soft drinks, together with caries in TT and the presence of MIH, were the variables associated with the presence of caries in the PFM [23].

Since there is no single model that aids in establishing the caries risk in teenagers, from the point of view of public health, it is necessary to use caries predictors that are cost-effective [24]. The importance of identifying caries risk factors prior to the eruption of PFMs in follow-up studies that include social and cultural aspects, together with dietary habits and the presence of caries in TT or MIH in PFM, justifies the present work. Our aim was to evaluate the risk factors included in the DCH and those factors’ effect on the appearance of caries in PT, and particularly in the PFM, seven years after their eruption.

## 2. Material and Methods

The present retrospective study was carried out following the principles of the Declaration of Helsinki, and was approved by the Ethics Committee of Investigation (Universitat de València, Valencia, Spain, procedure number: H1454270187226). Authorization for access to the information contained in the dental clinical history was provided by the Department of Health.

By means of the Population Information System (PIS), all children born in 2005 were listed, recording a total of 983 children. Inclusion criteria were as follows: children born in 2005, available information in their DCH before the eruption of the PT, and regular attendance to an oral health community program during a period of seven years after the eruption of their PFMs. Parents or caregivers of children who met the inclusion criteria were informed and asked to sign an informed consent to use the children’s dental clinical history data. DCH data compilation was carried out by an individual examiner authorized to access the information between January and March 2018. Information was registered in a database, excluding any element which could identify the participants. Consecutive numbers were assigned to each of the children enrolled in the study, without any reference to data which could identify them.

All of the participants in the study were included in an oral health community program (Valencia region, Spain), which covers all children between 0–14 years old and involves specific and non-specific measures for the promotion and prevention of oral health, as well as surgical procedures and restorative measures for PT.

The clinical examination, risk factor evaluation in the first visit, and seven-year follow-up were carried out by an individual examiner, different from the examiner who carried out the data collection.

The following variables were included:

- Age;
- Sex;
- Parental or primary caregiver caries experience 6 months prior to the inclusion of the child in the study;
- Educational level of the mother or primary caregiver;
- Dietary habits (daily sweets, pastries, snacks, or soft drinks);
- Toothbrushing frequency with fluoridated toothpaste;
- Routine medications;
- Systemic disease;
- Decayed and filled TT (df-t) at the start and at the end of the follow-up period;
- Decayed, missing, and filled PT (DMF-T) at the end of the follow-up period, and specifically in the PFM (DMF-M);
- Presence of MIH.

For data collection, every child enrolled in the study was clinically examined, and the presence of carious lesions was evaluated after drying teeth, using a dental mirror and a WHO/CPI/PSR probe, according the recommendations of ICDAS criteria for measuring dental caries [25]. Cavitated carious lesions (International Caries Detection and Assessment System (ICDAS) II codes 3–6) were differentiated from non-cavitated lesions (ICDAS II codes 1–2). MIH was evaluated following the criteria of the American Academy of Pediatric Dentistry (AAPD), proposed by Weerheijm et al. [26]. Bitewing X-rays were performed when the interproximal surfaces of molars or premolars were not accessible to visual examination.

Caries index scores were calculated for both TT and PT, considering only cavitated lesions or both cavitated and non-cavitated lesions. The specific caries index for the PFM was also evaluated. The following variables were recoded: educational level of the mother was recoded into two categories ((1) no education or primary education/(2) secondary or higher education); regular consumption of cariogenic food was renamed as “cariogenic diet” and recoded into two categories ((1) ≤2 intakes per day or (2) >2 intakes per day). Toothbrushing frequency was also recoded ((1) <1 per day or (2) ≥1 per day).

Independent variables were compared with dependent variables (DMF-T and DMF-M), considering cavitated lesions exclusively or including both cavitated and non-cavitated lesions, using Student’s *t*-test. Finally, variables significantly associated with dependent variables in the bivariate analysis were evaluated by Poisson regression with robust variance analysis. Statistical significance was considered at *p* < 0.05.

For statistical analysis, SPSS v25.0 program (SPSS Inc., Chicago, IL, USA) was used.

## 3. Results

From the 983 children born in 2005, after applying inclusion and exclusion criteria, the number of children enrolled in the study was 278 (28.28%). The mean age of the sample, after seven years of follow-up after the eruption of the PFM, was 12 years ± 7 months, and 50.7% were females. The df-t index (temporary decayed and filled teeth due to caries) was calculated in the first visit, considering only cavitated lesions (ICDAS II codes 3–6) or including non-cavitated lesions (ICDAS II codes 1–2) [27]. The obtained scores were 1.39 ± 2.46 and 1.47 ± 2.50, respectively.

After seven years of follow-up, DMF-T index was 0.60 ± 1.12 when only cavitated lesions were considered, and 1.18 ± 1.86 when both cavitated and non-cavitated lesions were included. The percentage of children without caries in their PT was 69.8% if only cavitated lesions were considered, and 58.6% if both cavitated and non-cavitated lesions were considered. With the same criteria, values referred to DMF-M were 0.58 ± 1.02 and 1.04 ± 1.48, and the percentage of caries-free children was 69.1% y 58.6%, respectively.

The distribution of children enrolled in the study, categorized according to mean and standard deviation of DMF-T and DMF-M indices and their relationship with the study variables, is represented in Table 1. It can be highlighted that the presence of parental caries in the 6 months prior to the inclusion in the study had a significant association with DMF-T and DMF-M indices, considering both cavitated and non-cavitated lesions. However, when considering both cavitated lesions exclusively or cavitated and non-cavitated lesions together, variables associated significatively with DMF-T and DMF-M index were the educational level of the primary caregiver, tooth brushing frequency, cariogenic diet, a score of >0 in the df-t index, and the presence of MIH both in PT and in the PFM.

Assessing the different food groups considered in the study, it can be determined that all cariogenic food groups were associated with higher caries indices, whether only cavitated lesions were considered or if incipient lesions were also included; the exception is in the case of industrial pastries, in which the differences were only significant if both cavitated and incipient lesions were included (Table 2).

Variables significantly related to caries in PT or in PFM in the bivariate analysis were included in a Poisson regression model (Table 3). All variables were significantly correlated to the DMF-T or DMF-M index, except for parental caries experience.

When the variables related to cariogenic food groups were included in the regression model and associated with caries index, sweets and soft drinks were the two food groups which showed a significant association (Table 4).

## 4. Discussion

The present retrospective, longitudinal cohort study covers a seven-year follow-up period after the eruption of the PFM, and provides information concerning the risk factors associated with the occurrence of carious lesions in PT, and particularly in the PFM, in children who attended a community dental program in the Valencia region (Spain).

Patients enrolled in the study are representatives of the entire social spectrum, as any child, from birth to 14 years of age, will attend one of the aforementioned community programs. All participants follow an oral health monitoring program consisting of oral hygiene instructions, topical high-concentration fluoride applications, sealing of pits and fissures (according to their individually identified caries risk), and restoration of permanent teeth, if necessary.

Caries prevalence in TT at 5–6 years of age, as well as the prevalence in the PT after seven years of follow-up (12–13 years of age), was similar to that reported in the national epidemiological study conducted in 2015 [9], as well as that of the Valencia region (Valencia, Spain) in 2018 [10], which shows that data from the present study can be extrapolated to the rest of the population. The distribution of the disease showed an asymmetric trend (DMF-T > 0 in 30.2%), as was also shown in national and regional studies in Spain [9,10], and as reported in the literature [28].

Different models have been used to evaluate the risk of caries development. The most used in clinical practice are CAMBRA (Caries Management by Risk Assessment) and Cariogram (complete or excluding data relative to bacterial levels, salivary flow, and buffer potential). The majority of studies regarding children report a significant association between the “high-risk” category, assigned using these models, and the present or future state of caries development [29,30,31]. Nonetheless, the predictive potential of the different models used to evaluate the risk of caries applied to the same population are different, both in longitudinal and cross-sectional studies [30,32]. In addition, a systematic review carried out by Cagetti et al. affirms that the evidence in relation to the different available models to predict the risk of caries is limited; however, the models may be useful for clinical practice [33]. Factors included in this study are those that are commonly considered in the different models for the evaluation of the risk of caries in clinical practice.

PFMs are fundamental teeth for the harmonious development of the dental arch, and their loss due to dental caries has adverse effects on occlusion [22]. Due to the age at which they erupt and their eruptive position, they have a high susceptibility to develop early carious lesions. For this reason, our primary aim was to assess the occurrence of carious lesions in PFMs within seven years from their eruption [34].

A total of 65.4% of the children with caries in their TT at the beginning of the study developed carious lesions in their PT within seven years, while only 27% of those without caries in their TT developed caries in their PT. In the same way, the caries index scores were significantly higher in the group of children with caries in their TT (Table 1). Therefore, not only the prevalence, but also the mean number of affected teeth presented by each child were higher in those who had experienced caries in their TT. Numerous studies have demonstrated that the presence of caries in TT is the best predictor of the development of caries in PT, particularly in the PFM [35,36,37,38]. Some studies even report that the presence of early childhood caries (ECC), whether treated or not, is a determinant factor for the development of caries in PT, specifically in the PFM [39].

When assessing the association between the presence of MIH and caries index scores, it was found that molars with MIH developed caries in 57.8% of the cases. In addition, DMF-T and DMF-M index scores were significantly superior in the MIH group compared to the cases in which there was no MIH. Similar findings have been reported in a systematic review, in which the probability for the development of caries in children with MIH was described to be between 2.4 and 4.6 times higher than in children with no MIH [40,41]. Using the Poisson regression model, MIH was found to be a risk factor for the development of caries in PT and in the PFM. The findings obtained indicate that special attention needs to be paid to the different pathological conditions that are frequently associated with MIH before the eruption of the PFM. Scientific literature indicates that patients with MIH require stricter preventive measures, with restorative treatments being up to ten times more frequent than in patients without MIH, even in cases in which risk factors are not present [42]. However, premature elimination of the affected area is not justified [43].

Among the modifiable risk factors related to lifestyle, such as dietary habits, the present study assessed the association between caries experience and cariogenic food consumption. An intake of food with cariogenic potential >2 times a day was associated with a prevalence of caries in the PT of 52% of the subjects. A lower intake was related to a prevalence of 28.9%. In the same way, DMF-T and DMF-M index scores were significantly higher in children with a cariogenic diet of >2 times a day. Specifically, the consumption of soft drinks and sweets showed a significant association in the Poison regression analysis, reporting caries in PT and in the PFM in all situations evaluated. Comparable results have been obtained in similar studies [44,45]. Consumption of soft drinks, such as sodas and packaged juices, has increased among children and adolescents in the last decades. This constitutes not only a problem for oral health, but also for health in general, both because of its high sugar content and low pH [46]. Therefore, parental and patient education regarding the harmful effects of these drinks is necessary.

In the present study, it was found that toothbrushing with fluoridated toothpastes at least twice a day was associated with significantly lower values of DMF-T and DMF-M index scores, along with a lower caries prevalence. Toothbrushing, in combination with a fluoridated toothpaste, is possibly the most important factor to prevent the occurrence of caries [47] and to remineralize existing carious lesions [48]. Other studies, like that of Winter et al. [49], also found that both frequent toothbrushing and the presence of fluoride in toothpaste were associated with a decrease in the incidence of carious lesions. Regarding fluoride concentration, the use of a 1000 ppm F^-^ paste is recommended [50]. In our study, the concentration used by the participants could not be determined precisely. This was due to the fact that many of the participants were unaware of the fluoride concentration of the toothpaste they used.

Other forms of fluoride self-application, such as daily or weekly use of fluoride-containing mouthwashes, were also evaluated. Of the participants, 4.3% reported using mouthwash. Those who did not use such products showed higher scores in caries indices. However, this association was not significant. Scientific evidence regarding the use of fluoridated mouthwashes is inconclusive, as shown in the systematic review by Marinho et al. [51].

In patients who suffered from a systemic disease, or in those who were routinely taking medications, caries index scores in PT were higher than in those who did not, although a statistical significance was not reached. This can be attributed to the low percentage of patients included in the study with both criteria.

Socioeconomic aspects cannot be ignored when analyzing predictive factors for caries development [52,53]. In the present study, all social strata were eligible, as it is a study carried out within the framework of a public oral health program. Evaluated factors were the educational level of caregivers and the parental caries experience in the 6 months prior to initial data collection. In this way, it was found that caries index scores, both in PT and in the PFM, were higher in children whose parents lacked education or only possessed primary education. This could be explained by the fact that the low educational level is associated with insufficient understanding, awareness, and poor knowledge about oral health [23,24,36,54]. These indices were also higher in children whose parents had experienced new carious lesions a few months before the first visit, compared to those who did not report caries in that period of time. However, this variable lost its significance when included in the regression model.

In brief, the results of the present study show that the experience of caries in TT, the presence of MIH, a cariogenic diet (especially soft drinks and sweets), toothbrushing frequency, and parental educational level were associated with a higher prevalence of caries and superior caries index scores in PT and in the PFM, seven years after its eruption. With the exception of the educational level of the primary caregiver, the rest of factors analyzed were also associated with caries development in the five-year follow-up study carried out by our research group [23]. This demonstrates that the risk factors present in the first years of age continue to have an important role in the development of caries later in life.

Based upon the results found in this study, and from the point of view of the clinical and dental public health application, implementing oral health education programs targeting pregnant women, parents, caregivers, and children seems necessary. In addition, periodic oral examinations should be carried out to ensure the early diagnosis of carious lesions and developmental defects of enamel, particularly MIH in the PFM.

## Figures and Tables

**Table 1:** Relationship between explanatory variables and response variables at seven years of follow-up.

			DMF-T C	DMF-T C + NC	DMF-M C	DMF-M C+ NC
		*n* (%)	M ± SD	M ± SD	M ± SD	M ± SD
**Initial df-t C**	df-t = 0	174 (62.59)	0.32 ± 0.75	0.63 ± 1.30	0.32 ± 0.75	0.56 ± 1.12
	df-t > 0	104 (37.41)	0.95 ± 1.13 **	1.14 ± 1.32 *	0.91 ± 1.06 **	1.09 ± 1.27 *
**Initial df-t C + NC**	df-t = 0	169 (60.79)	0.31 ± 0.76	0.57 ± 1.22	0.31 ± 0.76	0.51 ± 1.08
	df-t > 0	109 (39.21)	0.96 ± 1.11 ***	1.13 ± 1.29 **	0.91 ± 1.04 ***	1.09 ± 1.24 **
**MIH in PT**	absence	214 (76.98)	0.48 ± 1.06	1.13 ± 1.95	0.45 ± 0.90	0.93 ± 1.46
	presence	64 (23.02)	1.00 ± 1.23 ***	1.36 ± 1.48 *	1.01 ± 1.27 ***	1.40 ± 1.52 **
**MIH in PFM**	absence	226 (81.29)	0.49 ± 1.07	1.17 ± 1.96	0.47 ± 0.92	.99 ± 1.50
	presence	52 (18.71)	1.06 ± 1.23 ***	1.23 ± 1.35	1.08 ± 1.27 ***	1.25 ± 1.38 *
**Cariogenic diet**	≤2 daily	128 (46.05)	0.36 ± 0.83	0.73 ± 1.44	0.38 ± 0.84	0.70 ± 1.34
	>2 daily	150 (53.95)	0.80 ± 1.29 **	1.56 ± 2.08 ***	0.76 ± 1.12 **	1.33 ± 1.54 **
**Routine medications**	absence	259 (93.17)	0.59 ± 1.14	1.20 ± 1.89	0.58 ± 1.03	1.06 ± 1.50
	presence	19 (6.83)	0.63 ± 0.96	0.89 ± 1.24	0.58 ± 0.84	0.79 ± 1.18
**Systemic disease**	absence	247 (88.85)	0.63 ± 1.15	1.25 ± 1.91	0.60 ± 1.02	1.08 ± 1.49
	presence	31 (11.15)	0.32 ± 0.79	0.65 ± 1.23	0.45 ± 1.03	0.74 ± 1.37
**Brushing frequency**	<1 daily	58 (20.86)	1.03 ± 1.61 **	1.82 ± 2.16 **	0.93 ± 1.25 *	1.57 ± 1.63 *
	≥1 daily	220 (79.14)	0.48 ± 0.90	0.94 ± 1.71	0.49 ± 0.93	0.90 ± 1.37
**Educational level of the mother**	illiterate/primary	115 (41.37)	0.83 ± 1.37 **	1.74 ± 2.21 ***	0.81 ± 1.18 **	1.48 ± 1.59 ***
	secondary/university	163 (58.63)	0.43 ± 0.87	0.79 ± 1.44	0.43 ± 0.86	0.74 ± 1.32
**Parental caries experience 6 months prior to inclusion**	absence	182 (65.47)	0.51 ± 0.96	0.91 ± 1.50	0.52 ± 0.96	0.87 ± 1.38
	presence	96 (34.53)	0.76 ± 1.37	1.69 ± 2.32 **	0.70 ± 1.11	1.36 ± 1.60 **

Abbreviations: mean (M) and standard deviation (SD) of decayed, missing, and filled permanent teeth (DMF-T) and DMF in the permanent first molar (DMF-M); MIH: molar incisor hypomineralization; C: cavitated lesions; C + NC: cavitated and non-cavitated lesions: * *p* < 0.05; ** *p* < 0.01; *** *p* < 0.001.

**Table 2:** Association between DMF-T and DMF-M indices at seven years of follow-up, with dietary factors.

			DMF-T C	DMF-T C + NC	DMF-M C	DMF-M C + NC
		*n* (%)	M ± SD	M ± SD	M ± SD	M ± SD
**Sweets**	absence	159 (57.19)	0.40 ± 0.88	0.70 ± 1.33	0.42 ± 0.91	0.69 ± 1.31
	presence	119 (42.81)	0.87 ± 1.34 **	1.82 ± 2.24 ***	0.81 ± 1.11 **	1.50 ± 1.58 ***
**Pastries**	absence	165 (59.35)	0.51 ± 1.13	0.86 ± 1.56	0.49 ± 0.97	0.80 ± 1.37
	presence	113 (40.65)	0.73 ± 1.10	1.65 ± 2.15 ***	0.72 ± 1.08	1.39 ± 1.58 **
**Eating between meals**	absence	158 (56.83)	0.46 ± 0.91	0.82 ± 1.44	0.47 ± 0.94	0.80 ± 1.37
	presence	120 (43.17)	0.78 ± 1.33 *	1.65 ± 2.21 ***	0.73 ± 1.10 *	1.36 ± 1.57 **
**Soft drinks**	absence	91 (32.73)	0.33 ± 0.76	0.69 ± 1.40	0.32 ± 0.73	0.64 ± 1.25
	presence	187 (67.27)	0.73 ± 1.24 **	1.42 ± 2.00 **	0.71 ± 1.11 **	1.24 ± 1.55 **

Abbreviations: mean (M) and standard deviation (SD) of DMF-T and DMF-M index scores. C: cavitated lesions. NC: non cavitated lesions. * *p* < 0.05; ** *p* < 0.01; *** *p* < 0.001.

**Table 3:** Variables included in the Poisson regression model at seven years of follow-up.

	DMF-T C	DMF-T C + NC	DMF-M C	DMF-M C + NC
**df-t C (df-t>0 (ref.) df-t = 0**				−1.03 (−1.29/0.77) *p* = 0.00 ***
**df-t C+NC (df-t>0) (ref.) df-t = 0**	−1.08 (−1.43/−0.73) *p* = 0.00 ***	−1.08 (−1.33/−0.83) *p* = 0.00 ***	−1.14 (−1.45/0.79) *p* = 0.00 ***	
**MIH in PT (ref. yes) no**	−0.88 (−1.19/−0.56) *p* = 0.00***	−0.28 (−0.53/−0.03) *p* = 0.03 *		
**MIH in PFM (ref. yes) no**			−1.04 (−1.38/−0.71) *p* = 0.00 ***	−0.37 (−0.65/−0.09) *p* = 0.01 *
**Cariogenic diet (ref. ≤ per day)** **>2 per day**	−0.44 (−0.80/−0.08) *p* = 0.02 *	−0.37 (−0.62/−0.12) *p* = 0.00 ***	−0.42 (−0.78/−0.07) *p* = 0.02 *	−0.35 (−0.61/−0.09) *p* = 0.01 *
**Toothbrushing frequency (ref. <1 times/day)** **≥1 per day**	0.54 (0.21/0.87) *p* = 0.00 ***	0.25 (0.01/0.49) *p* = 0.04 *	0.40 (0.06/0.74) *p* = 0.02 *	
**Educational level of the primary carer (ref. no education/primary) = secondary/higher**	0.21 (−0.13/0.54) *p* =0.22	0.37 (0.13/0.60) *p* = 0.00 ***	0.29 (−0.04/0.62) *p* = 0.09	0.37 (0.12/0.62) *p* = 0.00 ***
**No parental caries experience 6 months prior to inclusion (ref.: yes)**	0.04 (−0.29/0.37) *p* = 0.81	−0.21 (−0.43/0.02) *p* = 0.08	0.19 (−0.15/0.53) *p* = 0.27	−0.06 (−0.31/0.18) *p* = 0.62

Abbreviations: C: cavitated lesions; NC: non cavitated lesions; ref.: reference. * *p* < 0.05; ** *p* < 0.01; *** *p* < 0.001.

**Table 4:** Dietary variables associated with caries experience at seven years of follow-up.

	DMF-T C	DMF-T C + NC	DMF-M C	DMF-M C + NC
**Sweets (ref. yes) no**	−0.70 (−1.10/−0.30) *p* = 0.00 *	−0.74 (−1.04/−0.46) *p* = 0.00 *	−0.59 (−1.00/−0.19) *p* = 0.00 *	−0.62 (−0.92/−0.31) *p* = 0.00 *
**Pastry (ref. yes) no**	0.16 (−0.20/0.52) *p* = 0.39	−0.14 (−0.40/0.12) *p* = 0.28	0.03 (−0.34/0.40) *p* = 0.88	−0.15 (−0.43/0.12) *p* = 0.28
**Eating between meals (ref. yes) no**	−0.07 (−0.46/0.32) *p* = 0.74	−0.10 (−0.38/0.19) *p* = 0.51	0.04 (−0.35/0.44) *p* = 0.83	−0.01 (−0.30/0.29) *p* = 0.97
**Soft drinks (ref. yes) no**	−0.66 (−1.07/−0.25) *p* = 0.00 *	−0.46 (−0.75/−0.17) *p* = 0.00 *	−0.69 (−1.11/−0.27) *p* = 0.00 *	−0.47 (−0.77/−0.17) *p* = 0.00 *

Abbreviations: C: cavitated lesions. NC: non cavitated lesions. * *p* < 0.05.
